# Lactoferrin Supplementation during Gestation and Lactation Is Efficient for Boosting Rat Pup Development

**DOI:** 10.3390/nu14142814

**Published:** 2022-07-08

**Authors:** Anne Blais, Annaïg Lan, Alice Boluktas, Marta Grauso-Culetto, Catherine Chaumontet, François Blachier, Anne-Marie Davila

**Affiliations:** AgroParisTech, Université Paris-Saclay, INRAE, UMR PNCA, 91120 Palaiseau, France; annaig.lan@agroparistech.fr (A.L.); alice.boluktas@gmail.fr (A.B.); marta.grausoculetto@cea.fr (M.G.-C.); chatherine.chaumontet@agroparistech.fr (C.C.); francois.blachier@agroparistech.fr (F.B.); anne-marie.davila-gay@agroparistech.fr (A.-M.D.)

**Keywords:** lactoferrin, pup development, intestinal differentiation, bone metabolism, brain development

## Abstract

Lactoferrin (LF) is an iron-binding protein found at relatively high concentrations in human milk. LF, which is little degraded in the infant intestinal lumen, is known to stimulate the proliferation and differentiation of the small intestine epithelial cells. The present study was designed to evaluate in the rat model the effects of bovine LF (bLF) given to the mothers during gestation and lactation on the growth of the offspring. Female Wistar rats were randomly separated into two groups of animals that received from mating and during gestation and lactation a standard diet including or not including bLF (10 g/kg of diet). The pups’ growth was determined up to postnatal day 17 (PND17), and parameters related to lean and fat mass, intestinal differentiation, intestinal barrier function, bone mineral density, osteoblast activity, and brain development were measured. In addition, metabolites in pup plasma were determined at PND17. bLF was detected in the plasma and milk of the supplemented mothers as well as in the pup plasma. Although the body weight of the pups in the two groups did not differ at birth, the pups recovered from the supplemented mothers displayed an increase body weight from PND12 up to PND17. At PND17 in the bLF group, increased small intestine epithelial cell differentiation was detected, and colon barrier function was reinforced in association with increased expression of genes coding for the tight-junction proteins. Regarding bone physiology, improved bone mineral density was measured in the pups. Lastly, the plasma metabolite analysis revealed mainly higher amino acid concentrations in the LF pups as compared to the control group. Our results support that bLF ingestion by the mother during gestation and lactation can promote pup early life development. The potential interest of supplementing the mothers with bLF in the case of risk of compromised early life development of the offspring in the context of animal and human nutrition is discussed.

## 1. Introduction

The early postnatal period is a key phase of development. Important structural and functional adaptations are put in place to allow the digestion and absorption of nutrients. Gut maturation during infancy relies, to a large extent, on adequate nutritional support, with milk representing the sole food during the early stage of rapid development. The composition of milk progressively changes post-parturition to meet the changing and specific requirements of the suckling neonate. The early-life period is known to be a key determinant of adult health [1]. Environmental exposure, especially nutrition, during this period can have long-lasting effects. The maternal diet has been shown to exert a direct impact on the placental flow of nutrients during gestation and can modulate milk composition during lactation [2]. The role of milk on the first day is to provide not only nutrients and micronutrients for growth but also to allow effective protection against infection [3]. Indeed, the nutritional values of milk are related not only to nutrients and micronutrients such as carbohydrates, proteins, lipids, vitamins, and minerals, but also to other biologically active constituents such as growth hormones, enzymes, anti-microbial factors, and anti-inflammatory agents. Among these compounds, lactoferrin (LF), an 80 kD sialylated iron-binding glycoprotein of the transferrin family found in high concentrations in human breast milk [4], is one of the four major glycoproteins in human milk, with concentrations averaging ~7 g/L in colostrum and 2–3 g/L in mature milk [5]. However, its concentration in bovine milk is only one-tenth the amount in human milk. Bovine and human LF share 69% amino acid sequence homology and similar three-dimensional structures [6]. One of the most important physiological functions of LF is the modulation of immune functions and its involvement in the host defense response against a spectrum of bacteria, fungi, yeasts, viruses, and parasites [7,8,9,10]. In addition, LF consumption inhibits the formation of several toxic compounds through the inhibition of virus replication and attachment to the cells and enhances systemic immune functions [11]. Bovine (b) LF supplementation of human preterm infants has been shown to reduce the incidence of sepsis and necrotizing enterocolitis [12]. Maternal bLF supplementation has also been shown to stimulate fetal growth [13]. LF has been shown to dose-dependently stimulate the proliferation and differentiation of the epithelial cells of the small intestine [14]. Moreover, LF supplementation has been shown to increase mice jejunal villus height and the expression of several intestinal brush border membrane enzyme activities [15] and to enhance piglet gut development and immune function [16]. Recent studies using a model of preterm hypoxic–ischemic brain injury reported that bLF supplementation in pregnant rats has neuroprotective effects on the development and cognition of the immature rat brain [17,18]. bLF supplementation in pregnant women was also shown to promote iron absorption and cell growth [19].

We previously demonstrated in adult mice supplemented with bLF (10 g/kg of diet) that bLF is resistant to major proteolytic degradation in the intestinal lumen and is transported from the intestinal lumen to the bloodstream as an intact molecule with the expected molecular size [20]. Moreover, immunoreactive bLF can reach most animal tissues, where it may exert its functions including antimicrobial and immuno-regulating actionsFisher bis. In addition, bLF ingestion was correlated with decreased osteoclast and increased osteoblast activities, thus supporting a direct effect on bone metabolism [21,22]. 

LF, the hydrolysis of which is minimal in infant intestinal luminal content, expresses multiple beneficial health effects, especially on the healthy development of infants. The study of Itell et al. [23] supports the interest of bLF supplementation in infants as they demonstrated bLF absorption into the bloodstream. In that overall context, the aim of the study was to evaluate the absorption of bLF by the pup and to determine the molecular mechanisms underlying the effect of bovine LF, given to the mother during gestation and then lactation, on the growth of the newborn during the neonatal period. Female rats were fed with a standard diet with or without bLF (10 g/kg of diet) at a concentration shown to be efficient in both rats and mice. The effects of bLF supplementation on pup growth were evaluated up to 17 days after birth. LF supplementation was found to act on gene transcription profiling and on plasma metabolite concentrations and these modifications were associated with enhanced neonatal growth, body composition, intestinal barrier function, bone mineral density, and brain development. 

## 2. Materials and Methods

### 2.1. Experimental Design and Diets

Eight 6-week-old female and four male Wistar rats (HsdHan^®^: WIST, Envigo, Gannat France) were maintained under controlled housing conditions (22 + 1 °C, 12 h light/12 h dark cycle with lights on at 8:00 a.m.) with free access to food and water. After 1 week of habituation, dams were housed individually and mated for one week. From the day of mating, they were concomitantly randomized to the different diets, one including bLF (10 g/kg of diet) and one without LF, until the end of the experiment. We chose a concentration of 10 g/kg of diet because this concentration has already been shown to be effective in rats and mice [17,21]. We used a standard diet in which soy protein composed 20% of the energy; 10 g of soy protein was replaced by bLF for the lactoferrin group and by casein for the control group (Table 1). Animal experiments were conducted according to the European legislation on animal experimentation and validated and approved by the Ethics Committee on Animal Experimentation, INRA, Jouy-en-Josas and the French Research Minister (APAFIS# 7379-2016 1 00518442065). At birth, litters were standardized to 8 pups by prioritizing females. The Cont group of pups originated from the control group of mothers and the bLF group originated from mothers fed with a bLF-supplemented diet. Offspring were weighted daily and on postnatal day (PND) 17 pups were anesthetized by isoflurane inhalation. Blood was drawn by cardiac puncture and the pups were then immediately decapitated.

### 2.2. Body Composition

Body composition (fat mass and lean mass) and bone mineral density (BMD) of the entire body, lumbar spine, and the right femoral bone were measured at the end of the study, under anesthesia just before euthanasia, by DEXA, using a Lunar Piximus densitometer (DEXA-GE Piximus; GE lunar Corp., Madison, WI, USA). The stability of the device was controlled by measurement of a phantom before each session. Analysis of the obtained images was performed with the software provided with the device (Lunar Piximus v2.10; GE Lunar Corp.) using auto-thresholding. Body composition was also determined by dissection of the liver, spleen, kidneys, intestine, and colon. Four white adipose tissue pads (periovarian, retroperitoneal, mesenteric, and total subcutaneous), interscapular brown adipose tissue, and the carcass (muscle and bone) were removed and weighed. 

### 2.3. Milk Sampling

Milk sampling was conducted on each mother 14 days after pup birth. Three hours before the sampling the mothers were separated from the pups. They were anesthetized by isoflurane inhalation and oxytocin was intraperitoneally injected. Manual milk sampling was conducted. After collection of about 1 mL of milk, the mother was put back in a cage until her total awakening, then was put back with her litter.

### 2.4. Brain-Derived Neurotrophic Factors (BDNFs)

Briefly, as previously described by Chen et al. [24], about 100 mg of hippocampal tissue was homogenized in 1 mL of cold lysis buffer (50 mM 4-(2-hydroxyethyl)-1-piperazineethanesulfonic acid (HEPES) (pH 7.5), 2 mM EDTA, 50 mM NaCl, 2% Triton X-100, 1 mM dithiothreitol (DTT), and 1 mM phenylmethylsulfonyl fluoride (PMSF) in a prechilled glass homogenizer using 15 vertical strokes. The homogenates were transferred to a 2 mL tube and placed on ice for 1 h before centrifugation at 20,000× *g* at 4 °C for 20 min. Protein levels in the supernatant were quantitatively determined using a bicinchoninic acid (BCA) Protein Assay Kit (Thermo Scientific, Courtaboeuf, France). BDNF levels in the rat brain were determined using a sandwiched ELISA kit (ERBDNF Thermo Scientific). The absolute value of BDNFs in each hippocampal sample was expressed as ng/mg protein.

### 2.5. Biochemical Analysis

N-terminal propeptides of type I procollagen (PINP) and C-terminal crosslinking telopeptides of type I collagen (CTx) were measured by enzyme immunoassay in the plasma of pups according to the manufacturer’s instructions (Immunodiagnostic Systems, Bolden Business Park, UK). Bovine lactoferrin concentration was measured in the plasma, milk, and urine of the mothers and in the plasma of the pups using a Bovine Lactoferrin ELISA Kit (Bethyl Laboratories, Montgomery, TX, USA). Plasma TNF-alpha and IL-6 levels were determined using a sandwiched ELISA kit (Thermo Scientific, Courtaboeuf, France).

### 2.6. Ex Vivo Intestinal Permeability Measurements

To assess potential changes in the intestinal barrier function, electrophysiological and epithelial permeability measurements were performed in Ussing chambers (EasyMount, Physiologic Instrument Inc., San Diego, CA, USA) as previously described [23] with the following modifications. Pup proximal colon and ileum samples were opened along the mesenteric line and mounted on an insert with an exposed area of 0.1 cm^2^. Mounted tissues were clamped at 0 mV to record the short-circuit current (I_sc_, uA/cm^2^) and left to equilibrate 30 min before trans-epithelial electrical resistance (R_t_, ohm/m^2^) measurement. The trans-epithelial conductance (G_t_, mS/cm^2^) was calculated as 1/*R*_t_. To evaluate paracellular permeability, fluorescein isothiocyanate (FITC)-dextran 4000 (FD4, Sigma-Aldrich, Saint-Quentin-Fallavier, France) was added soon after tissue mounting to the mucosal side at a final concentration of 0.250 mg/mL. After 90 min, FITC fluorescence flowed in the serosal side chamber was measured with a spectrophotometer (TECAN Infinite 200 Pro). Intestinal response to 10 mM glucose applied at the mucosal side, measured as *I*_sc_ increase, was tested after tissue equilibration. Tissue viability at the end of each experiment was verified with carbachol (CCh, 10^−4^ M), applied at the serosal side of the tissue, looking for the activation of the calcium-dependent chloride secretion by the *I*_sc_ increase.

### 2.7. Total RNA Extraction and RT-qPCR Experiments

The samples were kept at −80 °C and they were unfrozen just before total RNA extraction. Total RNA was extracted using TRIzol reagent, after homogenization using a TissueLyser (Qiagen, Courtaboeuf, France), and RNA concentrations in samples were measured with a NanoDrop ND-1000 UV-Vis spectrophotometer. RNA integrity was checked by ethidium bromide staining. An amount of 0.4 μg of total RNA in a final volume of 10 μL was reverse transcribed using a high-capacity cDNA archive kit protocol (Life technology, Courtaboeuf, France). Real-time PCR was performed using a StepOne Real-time PCR (Applied Biosystems, Fisher scientific, Illkirch, France) and the Power SYBR Green PCR Master Mix (Applied Biosystems) as previously described [25,26,27]. Gene expression levels for each sample were normalized relative to HPRT with 2^−ΔΔCT^ calculation. 

### 2.8. Bone Characteristics

The left femur of each mouse was cleaned of muscles and dried overnight at 110 °C, weighed, then ashed at 550 °C for 48 h, and the weight of the ash was then evaluated. The difference between the dry weight and the ash gives an indication of the protein fraction in bone.

### 2.9. Plasma Metabolite Extraction

The plasma samples were prepared for the characterization of metabolite content. The plasma proteins were extracted by adding cold methanol in a 3:1 ratio for 30 min at 4 °C. Then, samples were centrifuged for 10 min at 14,000× *g* and dried using a Speedvac. Samples were reconstituted in water and vortexed for liquid chromatography-mass spectrometry (LC-MS). Sonication was applied thereafter.

### 2.10. GC-MS Analysis of the Plasma

The analysis of volatile compounds was carried out on an Agilent 7890A gas chromatograph (GC) equipped with a split–splitless injector. The GC was coupled to an Agilent 5977A mass spectrometer (MS) with a quadrupole type mass filter and an electronic impact ionization mode. The GC was equipped with an upstream Gerstel MPS2-XL sampler and with a liquid injection module allowing the analysis of samples. The parameters of the sample changer were as follows: an injection volume of 1 μL carried out in splitless mode at 250 °C. The volatile metabolites were separated on a DB-5 MS capillary column (30 m × 0.25 mm × 0.25 μm; Agilent J&W). The initial temperature of the oven was 140 °C, maintained for 5 min. The temperature was increased by steps of 4 °C/min until reaching 240 °C, then was maintained for 20 min; the transfer line was at 280 °C. The MS was used in scan mode with a solvent delay of 4.6 min; the mass range used to identify the metabolites was between 29 and 450 *m*/*z*.

### 2.11. LC-MS Analysis of the Plasma

Samples were analyzed by high-pressure liquid chromatography (HPLC)/mass spectrometry on an LTQ Orbitrap (Thermo Scientific). The injection volume was 5 μL. Reversed-phase chromatography was carried out on a 2.1 mm × 100 mm × 3 μm Prevail C18 HPLC column from Grace. The column was maintained at 40 °C and elution was perormed using the gradients. An eluent flow rate of 200 μL/min was used and mass spectrometric data were collected over the mass range of 150–2000 in the positive (ESI+) electrospray ionization mode on an Orbitrap mass spectrometer [28,29,30,31] with a resolution of 60,000. Calibration was performed using a mix of caffeine, MRFA peptide (*m/z* = 524.2649), and Ultramark 1621 (reference polymer for mass calibration). A quality control (QC) sample was prepared by combining equal aliquots from all samples that were analyzed [32,33]. This QC sample was injected at the beginning of the analytical run (10 injections), in order to condition the chromatographic column, and periodically throughout the run every 10 injections in order to assess the analytical variability. Tandem mass spectrometry (MS/MS) experiments were performed on features of interest to guarantee a higher level of certainty for their identification [34]. They were identified by comparing their accurate mass and MS/MS information to the Human Metabolome Database (HMDB).

### 2.12. Metabolomics Data Processing and Analysis

A multivariate data analysis was performed using the SIMCA software. Untargeted principal components analysis (PCA) was applied to check the quality of the analysis, to identify deviant sample (outliers), and to have a first overview on the quality of the data. For classification and for the search of biomarkers, orthogonal partial least square-discriminant analysis (OPLS-DA) was performed. These supervised analyses made it possible to observe whether there was a discrimination between the classes. Each point present on the S-plot is representative of a more or less discriminating metabolite for one of the two groups. In OPLS-DA, class separation is maximized in the predictive component (*x*-axis) and its orthogonal component (*y*-axis) expresses intra-class variability. Discrimination is all the more important when the covariance and correlation are high. From the S-plot and *m/z* indications of the discriminating metabolites, the chromatogram of the bLF or CONT sample makes it possible to target a peak according to the metabolite sought.

### 2.13. Statistical Analysis

Only the female pups from the two groups were used to minimize the differences between males and females. All data are expressed as means ± SEM. We performed the unpaired Student’s *t*-test and repeated-measures two-way ANOVA to determine the effects of the dam diet during gestation and the interactions between maternal diet factors. In the post weaning models, a random factor was added to include correlations between pups from the same litter. Pairwise comparisons were adjusted for multiple comparisons using a Tukey post hoc test. Statistical analyses were performed using R studio 1.1.383, and the differences between groups were considered to be significant at *p* < 0.05. 

## 3. Results

### 3.1. bLF Supplementation in Mothers Accelerates Pup Growth

**Figure 1:**
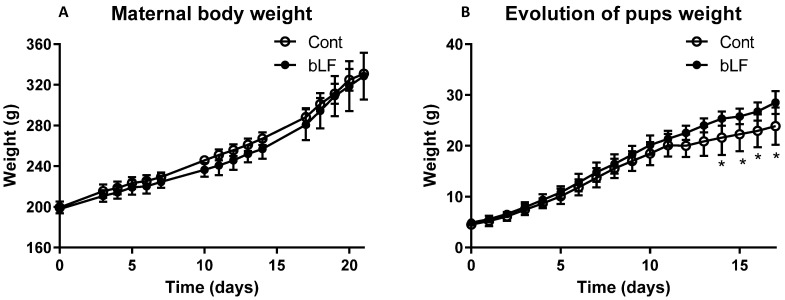
Effect of bLF supplementation on mother and pup body weight evolution. Maternal body weight during gestation (**A**). Pup weight evolution from birth to 17 days (**B**). Maternal bLF supplementation (bLF) or without supplementation (Cont). Data represented are means ± SEM (* *p* < 0.05; *n* = 16 pups per group). The Cont and bLF groups were compared by an unpaired two-tailed Student’s *t*-test.

**Figure 2:**
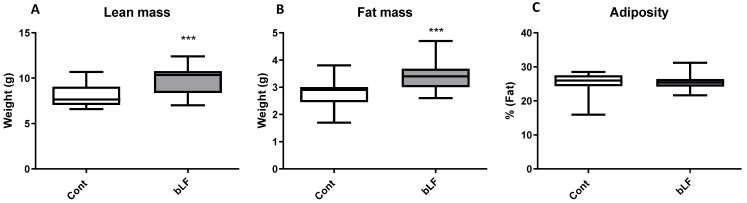
Composition of body weight at postnatal day 17 (DEXA values) of pups with maternal bLF supplementation (bLF) or without supplementation (Cont). (**A**) Effect of maternal bLF supplementation on the lean mass. (**B**) Effect of maternal bLF supplementation on fat mass. (**C**) Effect of maternal bLF supplementation on adiposity. Data represented are means ± SEM (*** *p* < 0.001; *n* = 16 per group). The control and lactoferrin groups were compared by an unpaired two-tailed Student’s *t*-test.

Maternal body weight throughout gestation was similar for both groups of mothers, supplemented or not with bLF (Figure 1A). At birth pup body weight of the LF group (4.91 g ± 0.29) was not significantly different from that of the control group (4.51 g ± 0.31). At day 12, pups of the LF group began to have a more important weight than the control group (*p* < 0.05) (Figure 1B). Pup DEXA body analysis showed that the increased pup weight induced by the maternal bLF supplementation was related to an increase of both the lean and fat mass (Figure 2A,B). However, the adiposity was similar in both groups (Figure 2C). This result supports that maternal bLF supplementation induced a more rapid growth of the pups. A detailed analysis of the body composition of the pups at postnatal day 17 is reported in Table 2. As the weight of the pups born from the mothers that received bLF was higher at day 17, the weight of the carcass, the different fat mass, and the organs was more important. These data confirmed the DEXA analysis. However, the relative weight (organ weight/body weight) of the liver and kidney were similar in both groups. Values of 0.0296 ± 0.0006 versus 0.0310 ± 0.0005 for the liver and of 0.0105 ± 0.0001 versus 0.0107 ± 0.0002 for the kidney were measured for the Cont and bLF groups, respectively, supporting the notion that bLF supplementation in the mothers exerts no major toxic effect on the offspring in this experimental model.

### 3.2. bLF Supplementation in Mothers Increases the Pup Brain Weight but Has no Effect on BDNF Expression

Although maternal bLF supplementation increased the pup brain weight at 17 days, in accordance with the increased body weight, we did not measure any effect of bLF on BDNF level and on BDNF gene expression (Table 3).

### 3.3. bLF Supplementation in Mothers Does Not Increase Pro-Inflammatory Cytokines to Any Detectable Level in Mothers and Pups

TNFα and IL-6 plasma levels were evaluated in mothers and pups. The levels of these cytokines were too low to be determined in pups. IL-6 level in the plasma of the mothers was also too low to be robustly measured. bLF ingestion by the mothers did not modify the TNFα plasma level in mothers, with such a level being at the limit of detection (4 ng/mL) (data not shown).

### 3.4. Supplementation of the Mothers with bLF Leads to the Presence of the Protein in Their Plasma and Milk, as well as in the Plasma of Pups

**Figure 3:**
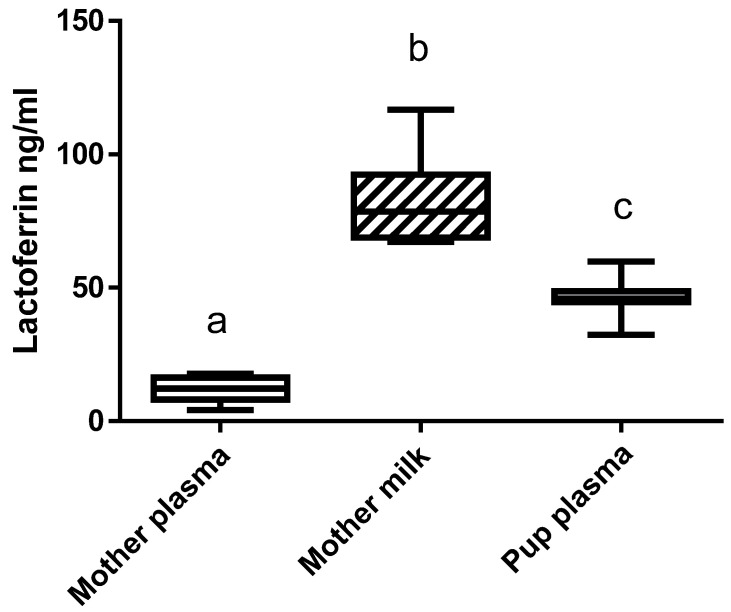
bLF concentration in the plasma and milk of the mothers supplemented with bLF and in pup plasma. Data represented are means ± SEM (groups with different letters are significantly different) (*n* = 8 for the mothers and *n* = 16 for the pups). Each group was compared with the others by a one-way ANOVA followed by a Tukey post hoc test. Means that are significantly different (*p* < 0.05) according to the Tukey multiple comparison test have different letters.

Immunoreactive bLF concentration in the mothers’ plasma and milk and in the blood of the pups fed by the mothers receiving bLF was evaluated (Figure 3). Immunoreactive bLF was found in the plasma and milk of the mothers supplemented with bLF. The amount of bLF found in milk was 6 times higher than in plasma. bLF was detected in the plasma of the pups fed by the mothers supplemented with bLF. As expected, no bLF was detected in the milk or plasma of mothers not supplemented with bLF. A preliminary analysis showed that ingestion of bLF did not have any significant effect on rat LF plasma concentration.

### 3.5. bLF Supplementation in Mothers Increases the Differentiation of Enterocytes in the Pup Small Intestine and Reinforces Colonic Epithelium Barrier Function

To assess the potential effect of maternal bLF supplementation on intestinal barrier function measured in the pups, several experiments were performed. Firstly, the transmural electrical parameters of the ileal intestine and proximal colon were measured in an Ussing chamber (Table 4). Maternal bLF supplementation did not modulate the FD4 permeability across the pup intestinal mucosa, either in the small or large intestine, thus indicating that the paracellular pathway of the gut barrier function was not altered by bLF ingestion. In the ileum of the pups, bLF ingestion did not modify to any significant extent glucose absorption, short-circuit current (Isc), and trans-epithelial conductance (Gt). The colon Isc of the pups was not modified by bLF ingestion, but Gt was significantly reduced by the supplementation, supporting the view that bLF ingestion reduced the conductance across the colon mucosa and increased the resistivity. Accordingly, the colon permeability was reduced in pups recovered from mothers supplemented with bLF. At 17 days after bLF ingestion, no modification of the ileal barrier function was recorded in the pups, indicating that the effect of bLF ingestion on the colon barrier function appears specific. 

**Figure 4:**
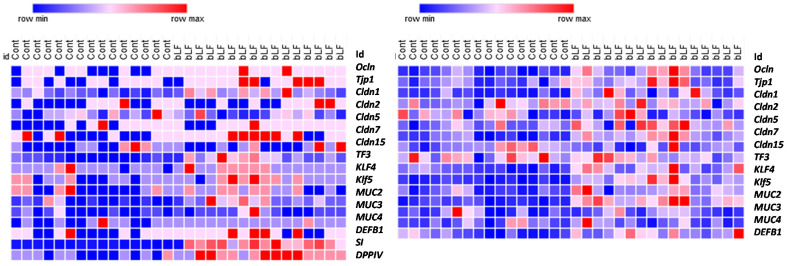
Heatmaps showing different levels of expression of gene coding for proteins involved in intestinal barrier function between pups recovered from mothers ingesting or not ingesting bLF. Color change from blue to red represents genes expression from low to high (16 pups were included in each group).

Heatmap representation of the transcript levels of several genes involved in the intestinal and colon barrier function and differentiation supports the view that bLF supplementation accelerated the intestinal maturation (Figure 4). Expression of the tight-junction proteins was more importantly increased in the colon than in the ileum in the pups recovered from the mothers receiving bLF supplementation (Table 5). Maternal bLF supplementation only increased occludin (*Ocln*) and claudin-15 (*Cldn*15) in the ileum; however, in the colon, *Ocln, Tjp1, Vil1,* and *Cldn2, 5, and 7* were increased. These data are in accordance with the epithelial barrier characteristics. The three goblet cell markers studied were increased in the colon, while only *Tff3* and *Klf* 4 were increased in the ileum. *Muc* 2, one of the major intestinal mucins, was increased in both the ileum and colon. The antimicrobial peptide *Defb*1 was increased in the pup small intestine but not in the colon. Lastly, an important increase of both sucrose isomaltase (*SI*) and dipeptidyl peptidase (*DPPIV*) gene expressions in the small intestine of the pups was measured following bLF ingestion, supporting that bLF accelerates enterocyte differentiation. This gene expression analysis revealed that bLF did not have a similar impact on the ileum and colon.

### 3.6. bLF Supplementation of Mothers Improves the Bone Mineral Density (BMD) in the Pups and Reduces Osteoclast Activity

**Figure 5:**
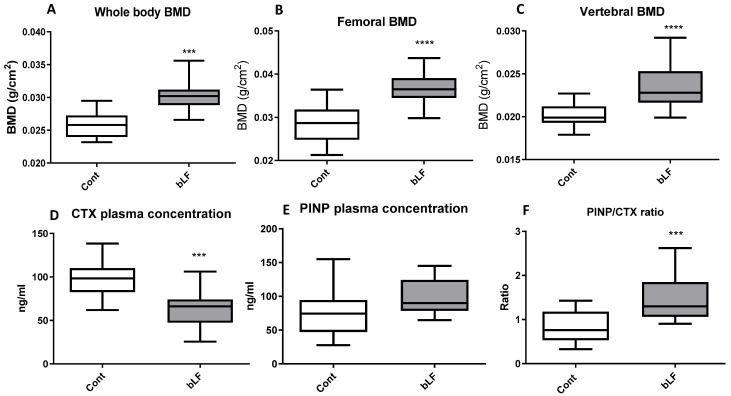
Effects of bLF supplementation on pup bone mineral density (BMD) and bone markers. Pups were recovered from mothers receiving the control diet (Cont) or the bLF-supplemented diet (bLF). (**A**) Whole body BMD. (**B**) Femoral BMD. (**C**) Vertebral BMD. (**D**) Plasma marker of bone resorption CTX. (**E**) Plasma marker of bone formation PINP. (**F**) PINP/CTX ratio. Data presented are means ± SEM (*** *p* < 0.001, **** *p* < 0.0001; *n* = 16 per group). The control and lactoferrin groups were compared by an unpaired two-tailed Student’s *t*-test.

After bLF supplementation in the mothers, the whole body, femoral, and vertebral BMDs were evaluated in pups at 17 days after birth. Figure 5 shows that maternal bLF supplementation increased pup whole body, vertebral, and femoral BMDs. To better characterize the bLF mechanism of action, bone remodeling markers were evaluated. Figure 5 shows that the CTX plasma concentration, a bone marker of osteoclast activity, was reduced in the pups fed by mothers supplemented with bLF. The PINP plasma level, used as a bone marker of osteoblast activity, was not significantly modified in pups when comparing the group of mothers supplemented with bLF with the group of mothers receiving no supplementation. The higher BMD reported for the pups fed by the bLF-supplemented mothers is correlated to an increase of bone formation as shown by the PINP/CTX ratio increase. This increased ratio is in accordance with the increased femur dry weight, collagen, and bone mineral contents (Table 6).

### 3.7. bLF Supplementation in Mothers Modifies the Metabolite Profile in Plasma Recovered from Pups

The impact of maternal bLF supplementation during gestation and lactation on the pup plasma metabolite profiles was determined at 17 days after birth for the control and the LF groups. The profiles were generated by two complementary approaches: GC-MS and LC-MS. The nature of the molecules generated belongs to the family of metabolites of the Krebs cycle, amino acids and derivatives, bile acids, phospholipids and lysophospholipids, sphingolipids, carnitines, and organic acids.

**Figure 6:**
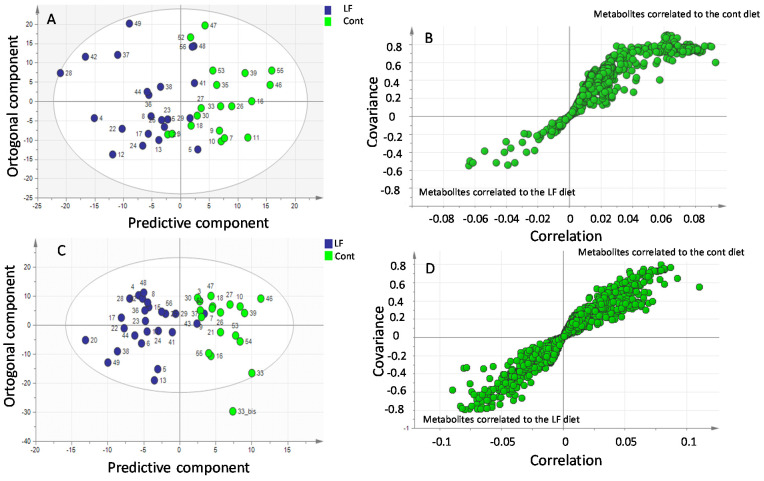
GC-MS and LC-MS multivariate analysis of metabolite profiles in plasma of pups recovered from mothers receiving bLF (LF) or mothers receiving no supplement (Cont). (**A**) GC-MS orthogonal projections of latent structures discriminant analysis (OPLS-DA) score plots of plasma metabolites in the control (green) and lactoferrin (blue) pups. LF vs. Cont (*p* < 0.01). (**B**) Loadings plots highlighting discriminant metabolites. (**C**) LC-MS orthogonal projections of latent structures discriminant analysis (OPLS-DA) score plots of plasma metabolites in the control (green) and lactoferrin (blue) pups. LF vs. Cont (*p* < 0.01). (**D**) Loadings plots highlighting discriminant metabolites.

The metabolite profiles in the plasma of pups originating from mothers supplemented or not with bLF were compared using a supervised multivariate OPLS-DA approach. We observed an excellent discrimination between the two groups. This model highlighted a discrimination according to the first two predictive components (65% of the total variation) (*p* < 0.01; R2X = 0.572; R2Y = 0.538; Q2 = 0.352) (Figure 6A).

A number of discriminating metabolites potentially involved in the separation between the two groups are presented in Figure 6B using an internally developed database. To determine the metabolites that were significantly increased in pups by maternal LF ingestion, we calculated the fold change (FC), which determines the quantitative change in the plasma of the LF pups compared to that of the control pups. Table 7 displays the identified metabolites significantly present at a higher concentration in the plasma of the LF pups. This table shows that many of the metabolites identified at higher concentrations in the LF plasma pups belong to amino acids. 

We also evaluated the impact of the litter on the metabolite profile in the plasma of the pups versus the diet given to them. To determine this possible impact, an intra-group comparison was necessary. The analysis showed no difference between the litters of the control group and those from the LF group (data not shown). This result indicates that modification of the metabolite profile in pup plasma is related to the diet and that the litter did not have any measurable impact on this parameter. 

Further analysis of the plasma samples by HPLC-MS generated 43 major classes of molecules. A clear difference was observed by LC-MS between the plasma of the pups originating from mothers receiving bLF supplementation and the pups originating from mothers not receiving bLF supplementation (69% of the total variation): *p*-value < 0.001 (R2X = 0.234; R2Y = 0.72; Q2 = 0.497) (Figure 6C). Many discriminating metabolites potentially involved in the separation between the two groups (Figure 6D) have been identified. Identification by LC-MS was carried out by two complementary approaches, namely, the use of our internal database (79 molecules referenced) and the use of external databases available on the internet (HMDB and MassBank). A list of candidates identified through those databases is presented in Table 8. Table 8 indicates an increase of many essential and non-essential amino acids in the plasma of the bLF pups. 

## 4. Discussion

The present study evaluated the effects of maternal bLF supplementation during gestation and lactation on rat pup development. The nutritional bLF supplementation showed no effect on gestational weight gain. Similar data have been reported by Somm et al. [13] when maternal bLF supplementation was given to healthy rats. The maternal bLF supplementation did not significantly increase the pup body weight at birth, but after 12 days, a significant increase of the pup weight was observed. Detailed analysis of the body composition indicated that pups fed by the mothers supplemented with bLF were growing more rapidly. In this study, we did not evaluate milk production, but it has been previously shown that oral administration of bLF can increase the milk production of gilts and the growth of their piglets [35], thus indicating that such an increased production is likely to explain in part the accelerated growth of neonates. However, as we showed that bLF was found not only in the milk of mothers supplemented with bLF but also in the pup plasma, this suggests that a direct action of bLF on several different tissues of the pups is also likely to partly explain the results obtained. 

Our data indicate an increase of the weight of all organs including the brain during bLF supplementation. The health benefits of lactoferrin on neurodevelopment and cognition have been previously reported [36]. Similar results have been previously shown in piglets [24]. However, we found no increase of the BDNF protein or corresponding gene expression. Similar results were observed in healthy rat pups in conditions of growth retardation. Other studies, in such a situation, have shown that bLF supplementation allows growth catch-up [13,37]. Interestingly, bLF supplementation was also shown to attenuate cerebral injury [17,18].

Evaluation of immunoreactive bLF showed that a higher concentration of bLF was found in the milk of the mothers than in their plasma, and that pups are able to absorb the bLF provided by the mothers. Using mice models, we previously showed that when mice ingested a diet supplemented with bLF, immunoreactive bLF was found in their plasma and in many different organs [20], thus indicating that bLF can be efficiently absorbed through the rodent intestinal epithelium. 

It has been shown that LF exerts a stimulating effect on the growth and differentiation of intestinal cells in vivo and in vitro [15,38,39]. As the development of rodent intestinal barrier function matures during the first 3 weeks of life, we studied the pup barrier functions at 17 days. To better characterize intestine development, we evaluated the expression of genes encoding proteins implicated in the tight junctions (TJs), notably claudins that are key regulators of the TJs, and other proteins such as goblet cell markers, innate immune system markers, mucins, and enzymes of the intestinal brush border that are all implicated in the intestinal barrier function and intestinal epithelium differentiation. Our data indicate that bLF increased the expression of most genes characterizing the intestinal functionality in pups. Barrier properties of the intestine depend on the TJs that are responsible for the junctional complex between the intestinal epithelial cells. Our results indicate that bLF ingestion significantly increased transepithelial resistance (TER) only in the colon. We chose to study claudin-1, -2, -5, -7, -8, and -15, taking into account that they are expressed in both segments (ileum and colon). Moreover, the expression of these claudins has been shown to be modulated as a function of time from birth to adult age [40]. Gene expression of *Ocln*, *TJP1*, and *Vil1* was increased in the colon, but only Ocln was increased in the small intestine of pups recovered from mothers ingesting bLF. However, we found significant modulation of the TER only in the colon. Moreover, only expression of *Cldn15* was increased in the ileum of pups, but *Cldn5* and *7* were increased specifically in the colon. Since the intestine is still very leaky up to 3 weeks, it is tempting to propose that the time point chosen may be too early to observe a significant effect of bLF ingestion by the mothers on TER in the pup ileum in a healthy condition. However, the beneficial effect of bLF ingestion has been reported in older piglets fed with bLF from 3 to 5 weeks. Indeed, in this study, bLF supplementation was shown to restore the intestinal morphology and to reduce the increased permeability in the situation of a restricted diet that provokes growth retardation [41]. As shown in Figure 4, most of the genes studied (tight junction proteins, goblet cell markers, mucins, innate immune system markers, and enzymes of the intestinal brush border) were more expressed in the pup intestine after supplementation of the mothers with bLF, supporting the view that such supplementation enhances gut development and immune function. In accordance with our results, similar data have been reported in a different context in postnatal piglets [16].

Since it has been shown that bLF can improve bone formation in mice [21,22] and in growing rats [42], the effect of maternal bLF supplementation on pup bones was evaluated. Our data indicate that bLF supplementation to the mother can play a key role in promoting the bone health of the offspring. LF supplementation has been shown to enhance bone mass in ovariectomized mice by a direct effect on bone metabolism [43]. In the present study, we found that maternal bLF supplementation increased bone mass in pups by decreasing the plasma level of the bone resorption marker CTX-1. Consequently, the PINP/CTX ratio was increased, suggesting that in young animals in which the bone formation is consequent and notably more important than the bone resorption, the main cellular target of bLF would likely be osteoclasts. A more important bone calcium content was observed in the present study, which suggests a reduced osteoclast activity. Our findings are consistent with the results of Xu et al. [42], showing that LF supplementation promotes bone health. 

The LC and GC-MS-based metabolomic approaches showed that the majority of metabolites detected in higher concentrations in the plasma of pups recovered from the mothers that received bLF supplementation were amino acids. At first glance, it is tempting to propose that a higher concentration of amino acids in plasma would favor protein synthesis in tissues by increasing the availability of the amino acid precursors. The GC-MS plasma metabolomic approach identified more specifically that L-arginine as well as ornithine were markedly increased in the pup plasma. Ornithine is an amino acid not present in dietary proteins that is mainly formed from L-arginine in various cells equipped with arginase activity [44,45].

Although the mechanisms that are at the origin of the increased concentration of arginine and ornithine in the pup plasma following the supplementation of mothers with bLF remain to be identified, it is worth noting that circulating arginine is well known to increase growth hormone secretion [46] and to further increase insulin secretion in response to increased glycemia [47].

Of note, the beneficial effects of arginine supplementation during gestation have previously been reported by Zeng et al. [48]. Interestingly, they reported that arginine can improve the survival of young rats and promote an increased size of litter at birth.

The LC-MS plasma metabolomic approach also confirmed an increase in amino acids in the plasma of pups recovered from the mothers supplemented with bLF. Among the essential amino acids that were increased, phenylalanine and tyrosine were found at higher concentrations in the LF group; thus, as mentioned above, they are potentially available for protein synthesis in the different tissues.

Non-essential amino acids, such as *trans*-4-hydroxy-L-Proline, were also measured in higher concentrations in the plasma of the bLF pups. Hydroxyproline is a non-essential amino acid found in collagen and a few other extracellular animal proteins. The *trans*-4-hydroxy-L-proline plays a crucial role in collagen synthesis, and higher concentrations of this amino acids can be correlated to the higher bone collagen synthesis reported in the bLF group. Betaine is supplied by the diet or can be obtained from the oxidation of choline. Betaine was also found to be increased in the plasma of the bLF pups. Betaine is believed to be important for embryo preimplantation and for its development during infancy [49]. Moreover, a study evaluating the concentrations of choline and betaine in human plasma during the second trimester of pregnancy found that the concentrations of these compounds are associated with early cognitive development in infants, supporting the view that choline or betaine supplementation may have beneficial effects in pregnant women during the first half of the pregnancy [50].

Betaine also plays a role in the metabolism of carnitine. Therefore, it is possible that the high level of this amino acid had an impact on the concentration of L-carnitine which was also measured in a higher concentration by LC-MS in the LF pups. Moreover, an increased level of L-carnitine during gestation and lactation was shown to increase the weight of the young at birth and during weaning compared to pigs under a controlled diet [51].

We also report an increased concentration for the LF group of two lipidic components of the cellular membrane: cardiolipins and ganglioside GM1. Cardiolipins are an important component of the inner mitochondrial membrane since they constitute more than 10% of the total lipid content [52]. Ganglioside GM1 is a component of cellular membranes in mammalian cells. It interacts with co-localized proteins in cellular membranes and modulates cellular functions such as apoptosis/survival, proliferation/differentiation, and immune cell function [53,54]. An optimal level of ganglioside expression is required to maintain membrane integrity [55]. These data are consistent with our previous work that reported that LF can decrease cellular apoptosis and increase intestinal cellular differentiation [15]. We also reported using microarray analysis that LF was able to induce in intestinal cells a complex set of modifications in gene expression corresponding to intracellular transducers, apoptosis-associated proteins, transcription factors, and extracellular cell signaling proteins [15].

## 5. Conclusions

Our work using an in vivo perinatal rodent model demonstrated the benefits of bLF supplementation during gestation and lactation on the post-natal development of pups, in association with biological effects on the small and large intestine, and on bone physiology. Indeed, the presence of bLF during gestation and lactation in the mother’s diet resulted in an increased concentration of this protein in the plasma and milk of the mother, and interestingly in the pup plasma, thus suggesting that bLF in the maternal milk is transferred through the pup intestinal epithelium to the circulating blood. Such an increased bLF plasma concentration in pups was associated with an accelerated small intestine epithelium differentiation, and a reinforced gut barrier function in the colon. In addition, bLF increased the bone mineral density in the offspring, an effect that was concomitant with a reduced osteoclast activity. Supplementation of the mothers with bLF resulted in a modified metabolite profile in the pup plasma, with several amino acids being notably increased. Although the mechanisms linking maternal bLF supplementation with the increased concentration of circulating amino acids remain to be identified, such amino acid availability likely favors protein synthesis in particular in pup tissues in the context of accelerated growth.

Further work is needed to test the potential interest of supplementing the mother with an extra-nutritional dose of bLF in the case of foreseeable risk of compromised post-natal development of the offspring in both the animal and human nutrition context.

## Figures and Tables

**Table 1:** Composition of the diets given to the control group of mothers (P20 Soy) or to the bLF-supplemented mothers (P20 Soy + bLF).

Ingredients (g/kg de Diet).	P20 Soy	P20 Soy + bLF
Soy protein ^a^	173	173
Lactoferrin ^b^	0	10
Casein ^c^	10	0
Corn starch ^d^	584	584
Sucrose ^e^	95	95
Soybean oil ^f^	40	40
Alpha cellulose ^g^	50	50
AIN 93M mineral mix ^h^	35	35
AIN 93M vitamins ^h^	10	10
Choline ^i^	2.3	2.3

^a^ MP Biomedicals, Irvine, CA, USA. ^b^ Armor Protéines, Saint-Brice-en-Coglès, France. ^c^ Ingredia, Arras, France. ^d^ Cargill, MN, USA. ^e^ CristalCo Pro, Paris, France. ^f^ Lesieur, Asnière-sur-Seine, France. ^g^ Prat Dumas, Couze Saint Font, France. ^h^ ICN Pharmaceuticals, Orsay, France. ^i^ Jefo Nutrition, Saint-Hyacinthe, Québec, Canada.

**Table 2:** Body composition of the pups recovered from mothers receiving the control diet or the bLF-supplemented diet. The body composition was determined at postnatal day 17.

Parameters	Cont	bLF	*p* Value
Total fat mass (g)	1.978 ± 0.078	2.3641 ± 0.127	0.0068
Internal fat (mg)	504.5 ± 26.2	514.9 ± 29.6	0.0108
Subcutaneous fat (g)	1.426 ± 0.096	1.667 ± 0.057	0.0241
Carcass (g)	6.993 ± 0.268	8.777 ± 0.207	<0.0001
Intestine (cm)	39.0 ± 2.4	48.4 ± 0.9	0.0005
Liver (g)	0.702 ± 0.045	1.018 ± 0.053	<0.0001
Kidney (mg)	265.9 ± 10.50	338.7 ± 9.2	<0.0001
Spleen (mg)	74.5 ± 3.7	103.7 ± 4.7	<0.0001
Pancreas (mg)	39.9 ± 3.7	59.2 ± 2.7	<0.0001

Data represented are means ± SEM (*n* = 16 per group). The control and lactoferrin groups were compared by an unpaired two-tailed Student’s *t*-test.

**Table 3:** Effect of bLF ingestion by the mothers on pup brain development.

Parameters	Cont	bLF	*p* Value
Brain weight (g)	1.27 ± 0.02	1.36 ± 0.01	0.0001
BDNF amount (ng/mg)	1.00 ± 0.04	1.09 ± 0.03	NS
BDNF expression	1.01 ± 0.21	0.84 ± 0.27	NS

Data represented are means ± SEM (*n* = 16 per group). The control and lactoferrin groups were compared by an unpaired two-tailed Student’s *t*-test.

**Table 4:** Epithelial barrier characteristics in ileum and colon of pups recovered from mothers receiving the control diet or the bLF-supplemented diet. The parameters were determined at postnatal day 17.

Parameters		Ileum			Colon	
	Cont	bLF	*p* Value	Cont	bLF	*p* Value
Isc (µA/cm^2^)	7.8 ± 2.7 (5)	5.3 ± 1.2 (8)	NS	15.3 ± 3.1 (5)	15.6 ± 1.0 (8)	NS
Rt (ohm/m^2^)	25.6 ± 11.6 (5)	25.0 ± 3.3 (8)	NS	36.5 ± 2.7 (5)	25.0 ± 3.3 (8)	0.0019
Gt (mS/cm^2^)	63.8 ± 16.5 (5)	47.8 ± 9.6 (8)	NS	27.4 ± 1.6 (5)	17.9 ± 1.1 (8)	0.0009
Δ Isc Glucose (µA/cm^2^)	17.8 ± 5.4 (5)	11.6 ± 1.9 (8)	NS	1.5 ± 0.8 (5)	3.0 ± 0.6 (8)	NS
FD4 (ng/mL)	137.5 ± 73.3 (4)	80.3 ± 24.8 (7)	NS	16.6 ± 10.6 (4)	48 ± 26.8 (8)	NS

Data represented are means ± SEM. The Cont and bLF groups were compared by an unpaired two-tailed Student’s *t*-test.

**Table 5:** Gene expression of the intestinal barrier function in ileum and colon of pups recovered from mothers receiving the control diet or the bLF-supplemented diet. The parameters were determined at postnatal day 17.

Parameters	Ileum			Colon		
	Cont	bLF	*p* Value	Cont	bLF	*p* Value
Tight-junction proteins						
*Ocln*	1.07 ± 0.12	1.77 ± 0.10	<0.0001	0.94 ± 0.11	1.63 ± 0.27	0.0003
*Tjp*1	1.02 ± 0.10	1.44 ± 0.25	NS	1.11 ± 0.15	2.77 ± 0.28	<0.0001
*Vil* 1	0.97 ± 0.16	0.80 ± 0.11	NS	0.96 ± 0.14	3.05 ± 0.27	<0.0001
*Cldn*1	1.14 ± 0.14	1.21 ± 0.18	NS	1.19 ± 0.19	1.26 ± 0.16	NS
*Cldn*2	1.17 ± 0.17	1.31 ± 0.36	NS	1.27 ± 0.30	2.98 ± 0.57	0.0113
*Cldn*5	1.14 ± 0.12	1.26 ± 0.10	NS	1.09± 0.12	2.16 ± 0.18	<0.0001
*Cldn*7	1.21 ± 0.17	1.32 ± 0.20	NS	1.13± 0.17	2.41 ± 0.35	0.0037
*Cldn*8	1.20 ± 0.19	1.32 ± 0.25	NS	1.21 ± 0.10	0.93 ± 0.10	NS
*Cldn*15	1.29 ± 0.26	5.06 ± 0.79	<0.0001	1.15 ± 0.14	1.07 ± 0.11	NS
Goblet cell marker						
*Tff*3	1.41 ± 0.14	1.91 ± 0.16	0.0240	1.17 ± 0.19	2.81 ± 0.38	0.0005
*Klf*4	1.20 ± 0.17	2.12 ± 0.24	0.0034	1.26 ± 0.26	4.66 ± 0.60	<0.0001
*Klf*5	1.29 ± 0.23	1.73 ± 0.23	NS	1.20 ± 0.26	3.02 ± 0.48	0.0030
Mucines						
*Muc* 2	1.18 ± 0.27	3.83 ± 0.66	0.0027	1.14 ± 0.14	5.11 ± 0.50	<0.0001
*Muc* 3	1.13 ± 0.22	2.06 ± 0.41	NS	1.23 ± 0.43	1.84 ± 0.30	NS
*Muc* 4	1.07 ± 0.19	1.19 ± 0.11	NS	1.27 ± 0.24	1.71 ± 0.29	NS
Antimicrobial peptide						
*Defb*1	1.14 ± 0.15	1.66 ± 0.17	0.0240	1.21 ± 0.17	1.48 ± 0.25	NS
Enzyme of the intestinal brush border						
SI	1.61 ± 0.17	34.83 ± 1.97	<0.0001			
DPPIV	1.13 ± 0.11	2.49 ± 0.25	<0.0001			

mRNA expression (expression arbitrary units) of the different genes (expression relative to *Hprt*). Values are means ± SEM (*n* = 16 per group). The control and lactoferrin groups were compared by an unpaired two-tailed Student’s *t*-test.

**Table 6:** Bone markers in pups recovered from mothers receiving the control diet or the bLF-supplemented diet. The parameters were determined at postnatal day 17.

Parameters	Cont	bLF	*p* Value
Femur weight (mg)	174.8 ± 5.3	180.7 ± 11.4	NS
Femur dry weight (mg)	61.06 ± 2.04	75.20 ± 2.07	<0.0001
Collagen (mg)	42.98 ± 1.28	49.92 ± 1.43	0.0009
Mineral (mg)	18.08 ± 0.82	25.32 ± 0.69	<0.0001
Mineral %	29.37 ± 0.54	33.72 ± 0.44	<0.0001

Data represented are means ± SEM (*n* = 16 per group). The control and lactoferrin groups were compared by an unpaired two-tailed Student’s *t*-test.

**Table 7:** GC-MS analysis of metabolites in plasma of pups recovered from mothers receiving the control diet or the bLF-supplemented diet. The parameters were determined at postnatal day 17. The metabolites presented in the table are those for which plasma concentrations are significantly different when comparing the control and the bLF-supplemented groups.

Metabolites Name	*m*/*z*	RT (min)	FC (bLF/cont)	Sub.Class
L-arginine monohydrochloride	274.165	9.502	2.934 ***	Non-essential amino acids and derivatives
Ornithine	186.091	10.414	2.087 **	Non-essential amino acids and derivatives
2,4 Pyridinylmethyl amino carbonyl cyclopropanecarboxylic	391.172	24.183	2.759 **	Non-essential amino acids and derivatives
Tetracosamethyl-cyclododecasiloxane	360.175	24.391	2.187 ***	Non-essential amino acids and derivatives
4-2-4 Dichlorophenyl 2 methyl perazinyl 1-yl benzonitrile	260.145	9.502	3.384 ***	Phenyl ethers
1,2 Bis (2 quinolylmethyl) ethylene	142.094	11.047	1.898 **	Thioethers
1,10 Dicarboxylic acid	391.172	23.306	2.691 ***	Organic compounds
Heptacosane	139.042	20.935	2.596 **	Alkanes
Tetradecanoic acid trimethyl ester	316.126	17.608	2.13 *	Lipids
Alpha-glucose	211.082	20.617	1.824 **	Carbohydrates

Data represented are means ± SEM (* *p* < 0.05, ** *p* < 0.01, and *** *p* < 0.001; *n* = 16 pups per group). The control and lactoferrin groups were compared by an unpaired two-tailed Student’s *t*-test.

**Table 8:** LC-MS analysis of metabolites in plasma of pups recovered from mothers receiving the control diet or the bLF-supplemented diet. The parameters were determined at postnatal day 17. The metabolites presented in the table are those for which plasma concentrations are significantly different when comparing the control and the bLF-supplemented groups.

Metabolites Name	*m*/*z*	RT (min)	FC (bLF/cont)	Sub.Class
Phenylalanine	166.087	4.296	1.361 ***	Essential amino acids
Tyrosine	182.082	1.956	1.369 **	Essential amino acids
*trans*-4-Hydroxy-L-Proline	263.104	1.331	1.519 **	Non-essential amino acids and derivatives
Trymethylglycin (betaine)	235.167	1.236	1.479 **	Non-essential amino acids and derivatives
2-Hydroxy-3-methylbutyric acid	324.932	1.079	1.745 **	Fatty acids and conjugates
L-Carnitine hydrochloride	162.112	1.173	1.205 *	Carnitines and acyl-carnitines
Murocholic acid (MCA)	373.29	21.746	4.742 *	Bile acids
Cardiolipin	702.865	1.064	2.780 ***	Membrane lipids
Ganglioside GM1	838.841	1.068	3.171 **	Membrane lipids
Phosphocholine chloride calcium	367.152	8.436	1.971 ***	Membrane lipid
1-Oleoyl-sn-glycero-3-phosphocholine	203.055	1.216	1.381 **	Membrane lipids
Dopamine	154.056	1.189	1.434 *	Biogenic amines
Xanthine	153.067	2.909	1.44 *	Imidazopyrimidines
Trimethylamine (TMA)	383.115	1.217	1.531 *	Methylamines

Data represented are means ± SEM (* *p* < 0.05, ** *p* < 0.01, and *** *p* < 0.001; *n* = 16 pups per group). The control and lactoferrin groups were compared by an unpaired two-tailed Student’s *t*-test.
